# Effects of a high‐sugar mixed meal on cerebrovascular haemodynamics in young, healthy versus middle‐aged adults with cardiometabolic risk factors

**DOI:** 10.1113/EP093238

**Published:** 2025-11-22

**Authors:** Krista S. Reed, Molly R. Crew, Abby M. Frescoln, Spencer M. Romanowski, Rudy J. Valentine, Wesley K. Lefferts

**Affiliations:** ^1^ Department of Kinesiology and Health Iowa State University Ames Iowa USA; ^2^ Department of Physical Therapy and Kinesiology University of Massachusetts Lowell Lowell Massachusetts USA

**Keywords:** arterial stiffness, hyperglycaemia, large artery haemodynamics, midlife, pulsatility

## Abstract

Consumption of a high‐sugar mixed meal (HSMM) increases both glucose and insulin and elicits mixed vascular effects, with reduced microvascular blood flow but increased conduit artery diameter and blood flow. In this study, we sought to examine: (1) whether an HSMM elicits vascular segment‐specific effects within cerebrovasculature; and (2) whether these responses differ between young healthy adults and middle‐aged adults with cardiometabolic disease (CMD) risk factors. Twenty‐one young, healthy adults (ages 18–39 years; 24 ± 6 years) and 20 middle‐aged adults (ages 40–65 years; 55 ± 5 years) with CMD risk factors (hypertension, obesity or dyslipidaemia) underwent cerebrovascular assessments before and at 30 and 60 min after a 930 kcal HSMM. Carotid and aortic stiffness and carotid artery characteristic impedance were assessed via ultrasound and tonometry. Middle cerebral artery mean velocity, pulsatility index and resistive index were assessed via transcranial Doppler. In comparison to baseline, common carotid artery diameter increased while stiffness decreased, contributing to a decrease in carotid artery characteristic impedance in both groups following the HSMM (*p* < 0.05). In comparison to baseline, middle cerebral artery mean velocity, pulsatility index and resistive index increased only in young adults (*p* < 0.05) following the HSMM. Differential increases in cerebral pulsatility among young adults compared with middle‐aged adults with CMD risk factors might reflect modest differences in upstream, extracranial artery pulsatile transmission following an HSMM, along with simultaneous, complex intracranial cerebrovascular responses that require further interrogation. Cumulatively, these data suggest that: (1) the cerebrovascular response to an HSMM might differ between segments of the extra‐ versus intracranial cerebrovasculature; and (2) middle‐aged adults with CMD risk factors might have altered cerebrovascular responsiveness to an HSMM compared with young, healthy adults.

## Abstract (highlights)

- **What is the central question of this study?**
  The aim of this study was to identify cerebrovascular responses to a high‐sugar mixed meal and the influence that the presence of cardiometabolic risk factors in middle‐aged adults has on this response, in comparison to young healthy adults.
- **What is the main finding and its importance?**
  We identified that pulsatile haemodynamic responses to a high‐sugar mixed meal might differ across segments of the intracranial versus intracranial cerebrovasculature. Furthermore, this response appears to be influenced by the combined presence of middle age and cardiometabolic risk factors.

## 1 INTRODUCTION

Meals disproportionately high in sugar and carbohydrates (HSMM) cause large increases in blood glucose and insulin levels and have been shown to elicit different effects from a standard meal (Loader et al., 2017; Patik et al., 2018; Šorli & Lenasi, 2023) and to result in mixed effects on vascular function (Gordin et al., 2016; Horton et al., 2020, 2021, 2022; Parker et al., 2020). The vascular responses to these HSMMs appear to be segment specific, with reduced microvascular blood volume, flow and velocity, but increased conduit artery diameter, blood flow and velocity (Parker et al., 2020). The effects of HSMMs on the cerebrovasculature, however, are unknown but highly relevant because these meals are routinely consumed in Western diets (Choi et al., 2021; Long et al., 2022) and might contribute to development of type 2 diabetes over time, a known risk factor of cerebrovascular dysfunction and disease (Alzheimer's Association, 2025). If cerebrovascular responses mirror those in the periphery, conduit artery vasodilatation might increase mean blood flow within the large cerebral vessels. This hyperaemic response might simultaneously and paradoxically increase the transmission of harmful pulsatile blood flow into the cerebrovasculature through decreased impedance and thus greater downstream transmission of forward wave energy into the sensitive cerebrovasculature. This, combined with increases in stiffness and blood pressure in response to acute increases in glucose and insulin (Horton et al., 2021; Kobayashi et al., 2021), might have implications for cerebral microvascular function, especially in those with cardiometabolic risk factors (e.g., type 2 diabetes, hypertension and obesity) that might already present with early manifestations of compromised vascular function (Li et al., 2024).

Midlife is accompanied by an increase in cardiometabolic disease (CMD) risk factors that might alter cerebrovascular responses to acute stressors (e.g., high‐sugar meals) (Gordin et al., 2016; Nowaczewska et al., 2019). Indeed, adults with cardiometabolic risk factors exhibit undesirable elevated arterial stiffness and decreased cerebral blood flow in hyperglycaemic states, indicating that they might be more vulnerable to potentially detrimental effects of an HSMM on cerebrovascular haemodynamics (Gordin et al., 2016; Li et al., 2024; Nowaczewska et al., 2019). Understanding whether middle‐aged adults with cardiometabolic risk factors exhibit a differential cerebrovascular response to HSMMs in comparison to healthy young adults might be important to gain a better understanding of how the cerebrovasculature might adapt over time to this repeated dietary stressor and accelerate cerebrovascular dysfunction in this at‐risk group.

The aims of this study were to examine: (1) cerebrovascular responses to consumption of a single HSMM; and (2) whether these cerebrovascular responses differed between young, healthy adults and middle‐aged adults with CMD risk factors. We elected to: (1) specifically target middle‐aged adults with CMD risk factors because this group is of high relevance for cerebrovascular health‐related research and is likely to consume such a meal as part of their regular diet; and (2) use young adults as the comparator group to maximize our ability to identify differential responses as part of this early‐stage research. It was hypothesized that: (1) a single HSMM would increase carotid artery blood flow and stiffness but decrease downstream middle cerebral artery (MCA) blood velocity and increase pulsatility, mirroring previous observations of segment‐specific divergent changes in the peripheral arteries (Parker et al., 2020); and (2) middle‐aged adults with CMD risk factors would have blunted increases in carotid blood flow and augmented increases in stiffness and MCA pulsatility in comparison to young, healthy adults without risk factors.

## 2 MATERIALS AND METHODS

### 2.1 Participants

Twenty‐one young, healthy adults (24 ± 6 years) and 20 middle‐aged adults (55 ± 5 years) with cardiometabolic risk factors were recruited for this study. Participants did not smoke or vape; had no history of stroke, cardiovascular event, concussion within the last 3 months, or pulmonary, renal, neurological or metabolic disease; were not taking hormonal replacement therapy; had a body mass index <40 kg/m^2^; were not currently pregnant; and did not have dementia (Montreal Cognitive Assessment score <21) or mild cognitive impairment (Montreal Cognitive Assessment score <26) or depression (defined as Patient Health Questionnaire‐9 score ≥10). Young, healthy adults were defined as individuals aged 18–39 years who were free from use of anti‐hypertensive and lipid‐lowering medications, without hypertension (blood pressure <130/80 mmHg) and not obese (body mass index <30 kg/m^2^). Middle‐aged adults with risk factors were aged 40–65 years, with at least one modifiable CMD risk factor, including stage 1 hypertension (blood pressure >129/79 and <140/90 mmHg), medicated hypertension (self‐reported anti‐hypertensive medication), dyslipidaemia (self‐reported physician diagnosis or use of cholesterol‐lowering medication, e.g., statin) and/or Class I/II obesity (body mass index 30–40 kg/m^2^). Of the 13 middle‐aged females, 10 were postmenopausal and three premenopausal. All participants provided written informed consent prior to study initiation, and all procedures were approved by the Iowa State University Institutional Review Board and conformed to the standards outlined in the *Declaration of Helsinki*.

### 2.2 Study design

Participants underwent an initial online screening to assess eligibility, followed by an in‐person screening to confirm and further assess eligibility. Eligible participants then completed 3 days of at‐home physical activity and dietary intake monitoring, followed by the experimental visit (a minimum of 72 h after the in‐person screening visit).

#### 2.2.1 Screening process

Participants completed an online prescreening survey to identify self‐reported exclusion criteria, followed by an in‐person informed consent and screening to reassess self‐reported exclusion criteria (e.g., health history) and measured exclusion criteria (e.g., body mass index, blood pressure, dementia and depression). Blood pressure was assessed during the in‐person screening visit to ensure eligibility using an automated oscillometric cuff (HEM‐907XL OMRON Healthcare, Inc., Lake Forest, IL, USA) following 5 min of quiet, seated rest with feet flat on the floor. Triplicate blood pressures were taken with 1 min of rest between measures while the technician remained outside the room. Values were averaged if two measures were within 5 mmHg or measures were repeated until two measurements achieved this criterion. Height was measured via stadiometer. Weight and body fat percentage were measured using lower‐body bioelectrical impedance (Tanita sc‐331s, Tanita Corp. of America, Inc.), with body mass index calculated as weight (in kilograms)/height (in metres)^2^.

#### 2.2.2 Experimental visit

Participants arrived for the experimental visit ≥3 days after the screening visit and were required to have abstained from caffeine and non‐essential medication (e.g., non‐steroidal anti‐inflammatory drugs, nutritional/dietary supplements, allergy medications) on the morning of testing, refrained from dietary supplements and alcohol for 12 h prior to assessment and refrained from exercise for 24 h prior to assessment. Menstruating females were tested during the early follicular phase of their menstrual cycle. Physical activity and dietary intake were monitored for 3 days prior to the experimental visit (see Supporting Information‐Methods). All outcome measures, detailed below, were collected at baseline after 10 min of quiet rest. Following initial baseline measures, participants consumed the HSMM (nine Hostess mini donuts and Nestle Nesquik Chocolate Milk) that was a total of 930 kcal (35.5 g fat, 129 g carbohydrates, 100.5 g sugar and 14 g protein) within a 10 min period. After consuming the meal, subjects were allowed to ambulate minimally (e.g., walk to the restroom), and all were resting supine for ≥10 min prior to post‐meal measures. Outcome measures were then re‐assessed at 30 and 60 min after consumption of the meal.

### 2.3 Outcome measures

#### 2.3.1 Blood glucose and insulin

Blood glucose and insulin were measured at baseline and at 30 and 60 min post‐meal consumption to account for changes in these values resulting from meal consumption. A trained phlebotomist collected venous blood samples at each time point via intravenous catheter, and the samples were centrifuged at 1455*g* for 10 min. Plasma insulin (ENZ‐KIT141, Insulin ELISA Kit; Enzo Life Sciences, Inc., Farmingdale, NY, USA) and glucose (GAHK 20, Glucose [HK] Assay Kit; Sigma‐Aldrich, St. Louis, MO, USA) were analysed in duplicate according to the manufacturer's instructions.

#### 2.3.2 Blood pressure

Brachial systolic and diastolic blood pressure were measured using an oscillometric cuff until consecutive duplicate measures were within 5 mmHg of each other. Carotid and brachial blood pressure waveforms were assessed via tonometry, with waveforms signal averaged across a 20 s epoch. Brachial pressure waveforms were calibrated to brachial systolic and diastolic cuff pressure. Brachial diastolic and mean pressures were derived from brachial pressure waveforms and used to calibrate carotid pressure waveforms to determine carotid systolic, diastolic and pulse pressure (systolic minus diastolic pressure).

#### 2.3.3 Large artery stiffness

Large artery stiffness was assessed at the aorta and common carotid arteries. Applanation tonometry (NIHem, Cardiovascular Engineering Inc.) was used to assess aortic stiffness as carotid–femoral pulse wave velocity (cfPWV) by capturing carotid and femoral pressures with ECG for simultaneous R‐wave gating. The cfPWV was calculated as the distance between carotid and femoral sites divided by the time lag (Δ*t*) between carotid and femoral waves (Townsend et al., 2015). Carotid stiffness was assessed below the carotid bulb using ultrasound (Aloka ProSound α7 and Arietta 70; Hitachi Healthcare Americas, Twinsburg, OH, USA) with a 7.5 to 10.0 Hz linear‐array probe, and carotid pressures obtained from contralateral carotid tonometry (Niki et al., 2002). Four sets of 7–10 distension waveforms traced using eTracking software were ensemble averaged to assess β‐stiffness, computed as: ln(systolic pressure/diastolic pressure)/[(systolic diameter minus diastolic diameter)/diastolic diameter]. Carotid diameter and pressure assessments ranged from being taken simultaneously (participants with stronger signals/easier pulse sites/carotid imaging) to within 5 min of each other (on participants with more challenging signal acquisition).

Carotid characteristic impedance was calculated in the time domain by dividing the peak derivative of carotid pressure by the peak derivative of flow, as has been done previously (Reed et al., 2024). The mean Doppler velocity signal from digitized common carotid Doppler audio waveforms was multiplied by the cross‐sectional area of the artery to calculate the common carotid volumetric flow rate. Common carotid artery cross‐sectional area was assessed during diastole (assuming a circular orifice) and measured longitudinally and proximal to the carotid bulb. Wave separation analysis was conducted by integrating carotid pressure and flow waveforms to derive the forward (Pf) and backward (Pb) pressure waves and the wave reflection index (RIx; ratio of Pb:Pf) (Torjesen et al., 2014).

#### 2.3.4 Cerebrovascular haemodynamics

Right MCA haemodynamics were assessed using transcranial Doppler using a 2 MHz transcranial Doppler probe secured to the temporal window via a headset (TOCM Neurovision, Multigon Industries, Inc.) following recommended insonation protocols (Lefferts et al., 2020). The MCA mean velocity and pulsatility index (PI) were calculated over eight, 7 s epochs across a 2 min period. Within‐day reliability for MCA PI and mean velocity are 3.5% and 2.7% in our laboratory. The MCA PI and conductance were calculated as [(systolic minus diastolic velocity)/mean velocity] and mean velocity/brachial waveform mean arterial pressure, respectively. Common carotid PI and mean velocity were calculated in a similar manner using pulsed‐wave Doppler (Arietta 70, Hitachi‐Aloka) over two, 12 s epochs. End‐tidal carbon dioxide (ETCO_2_) was measured for 2 min during the MCA insonation via mouthpiece and infrared spectroscopy (AGM100, MediPines Inc.).

Cerebrovascular reactivity was assessed with an adapted breath‐hold protocol (Klein et al., 2020) to assess changes in MCA mean velocity with increases in carbon dioxide. Data from the breath‐hold protocol were collected using ETCO_2_ via a mouthpiece and infrared spectroscopy (AGM100, MediPines Inc.) during MCA insonation in the supine position. The protocol consisted of eight paced breaths at 16 breaths/min, followed by a 20 s breath‐hold. This procedure was repeated four times. A slideshow was projected onto the ceiling to guide participants visually and audibly through the paced breathing and breath‐holds. The ∆ETCO_2_ was determined for each breath‐hold manoeuvre and was calculated by subtracting the average of the last two paced breaths before the breath‐hold from the peak partial ETCO_2_ response following the breath‐hold. Cerebrovascular reactivity was calculated as the change in MCA mean velocity relative to corresponding change in partial ETCO_2_ (∆MCAv/∆ETCO_2_) as absolute and (%∆MCAv/∆ETCO_2_) as relative responses.

### 2.4 Statistical analyses

This study did not use an a priori power analysis, but instead identified a sample of *n* = 40 based on the higher range of samples from other relevant acute meal challenge studies (*n* = 10–38) (Marley et al., 2017; Migdal et al., 2020; Parker et al., 2020; Patik et al., 2018). Data were analysed using the Statistical Package for the Social Sciences, v.25 (IBM, Inc., Armonk, NY, USA). Normality was assessed quantitatively using the Shapiro–Wilk test, with non‐normal data (carotid artery PI and cfPWV) logarithmically transformed successfully to meet normality assumptions. Descriptive characteristics between young and middle‐aged adults were compared using independent *t*‐tests for continuous variables and χ^2^ tests for categorical variables.

**FIGURE 1:**
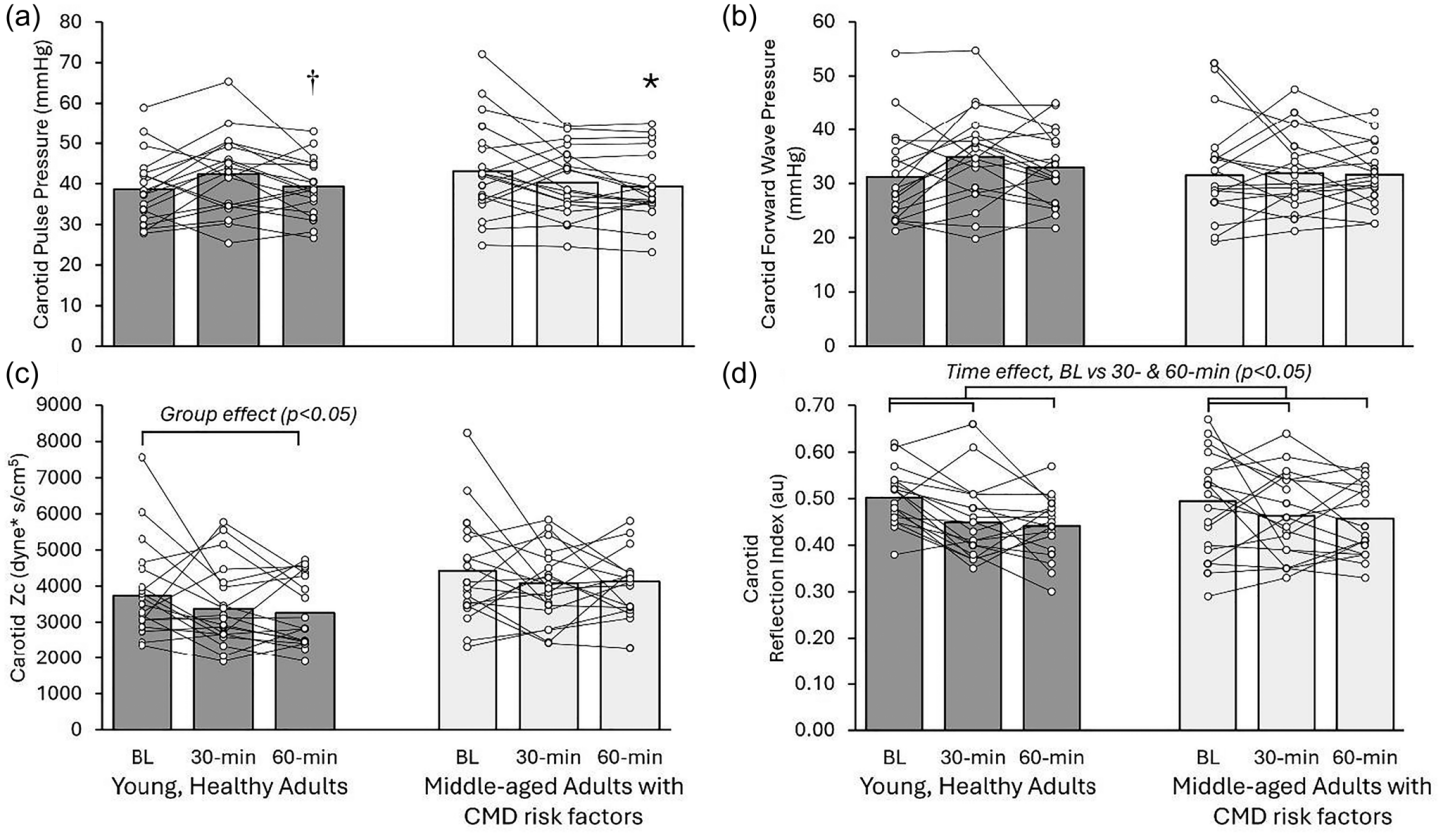
Changes in carotid pulse pressure (a), forward wave magnitude (b), characteristic impedance (c) and reflection index (d) in young, healthy adults (YA; *n* = 21) versus middle‐aged adults with cardiometabolic disease risk factors (MA; *n* = 20) at baseline and at 30 and 60 min following the high‐sugar mixed meal. Statistical effects were tested via repeated‐measures ANOVA. *p < 0.05 versus BL. †*p* < 0.05 versus within group at 30 min. Main effects are reported as *p* [partial η^2^]: (a) carotid pulse pressure (group 0.61 [0.01], time 0.048 [0.08], group × time 0.002 [0.15]); (b) carotid forward wave pressure (group 0.87 [0.00], time 0.27 [0.03], group × time 0.09 [0.06]); (c) carotid characteristic impedance (group 0.01 [0.15], time 0.04 [0.08], group × time 0.93 [0.00]); and (d) carotid reflection index (group 0.97 [0.00], time 0.005 [0.13], group × time 0.28 [0.03]). Abbreviations: BL, baseline; CMD, cardiometabolic disease; HSMM, high‐sugar mixed meal; MCA, middle cerebral artery; Zc, characteristic impedance.

**FIGURE 2:**
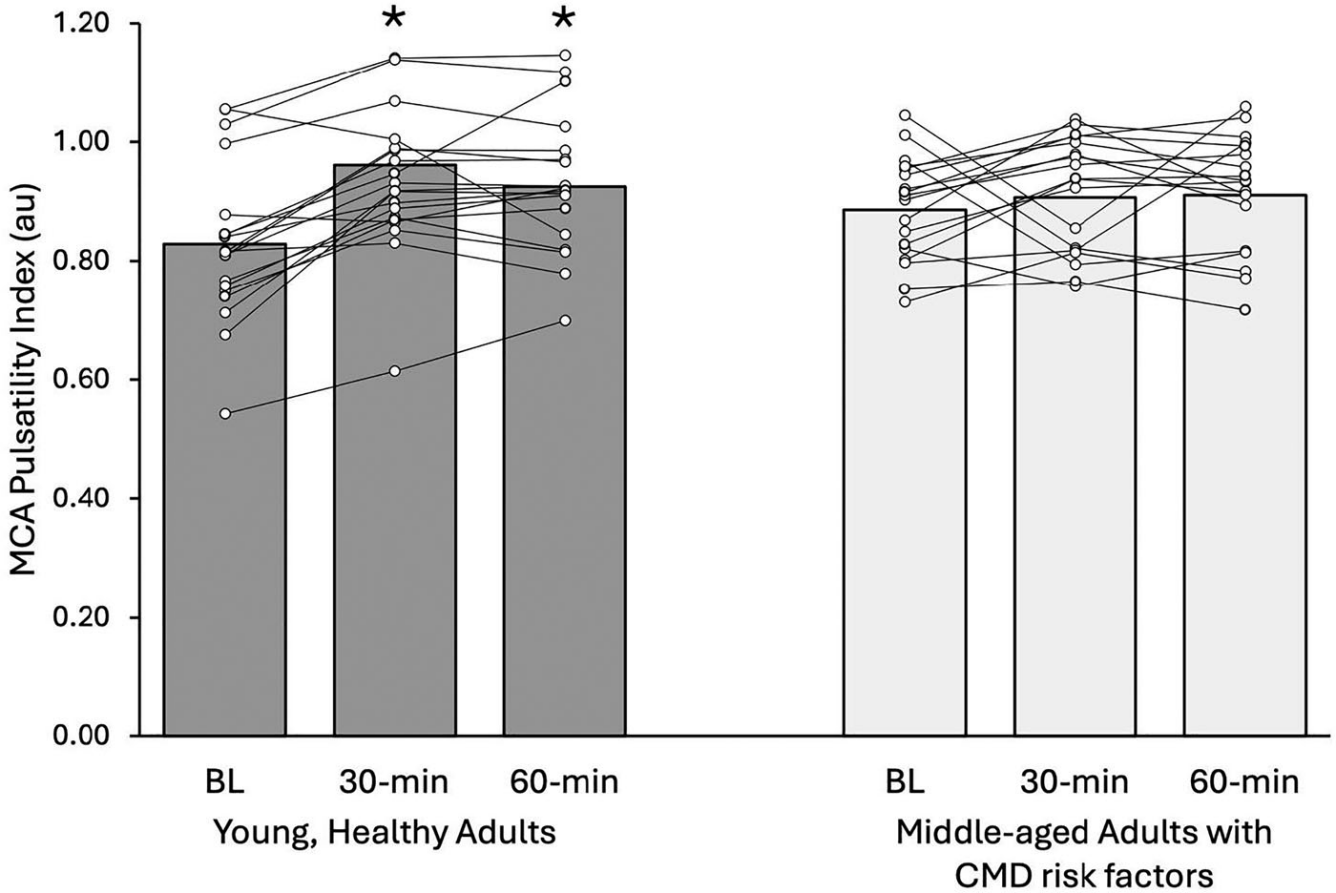
Changes in MCA PI in young, healthy adults (*n* = 21) versus middle‐aged adults with CMD risk factors (*n* = 18) at baseline and at 30 and 60 min following the HSMM. *p < 0.05 versus BL. Statistical effects were tested via repeated‐measures ANOVA. Main effects, *p* (partial η^2^): group 0.99 (0.001), time < 0.001 (0.22), group × time 0.01 (0.11). Abbreviations: BL, baseline; CMD, cardiometabolic disease; HSMM, high‐sugar mixed meal; MA, middle‐aged adult with cardiometabolic disease risk factors; MCA, middle cerebral artery; PI, pulsatility index; YA, young, healthy adult.

The effects of the HSMM on all outcome measures were assessed using a 2 × 3 [2 groups (YA, MA) × 3 times (baseline, 30, 60)] repeated‐measures ANOVA. Bonferroni‐corrected *post hoc* pairwise tests were used to explore significant group‐by‐time interactions further. Effect sizes for main effects are shown as partial eta squared (η^2^) along with *p*‐values. The η^2^ values represent the percentage variance in the dependent variable attributable to the independent variable, with 0.01, 0.06 and 0.14 representing small, medium and large effects, respectively. The Supplemental Results contains: (1) mean ± SD and statistical main effects for data presented in Figures 1 and 2 (Table S1); and (2) correlation matrices examining linear associations between HSMM‐induced changes in blood pressure, carotid diameter and large artery stiffness (Tables S2–S4).

## 3 RESULTS

Descriptive characteristics by group are displayed in Table 1. There were no significant differences between groups on any measures, except for age, body mass index and body fat percentage, which were significantly higher in the middle‐aged group compared with the young group.

**TABLE 1:** Descriptive characteristics of the study participants by group.

Characteristic	YA (*n* = 21)	MA (*n* = 20)
Age, years	24 ± 6	55 ± 5*
Sex, female, *n* (%)	12 (57)	13 (65)
Height, cm	173.2 ± 9.4	169.5 ± 10.3
Weight, kg	71.6 ± 14.3	81.8 ± 20.7
Body mass index, kg/m^2^	23.7 ± 3.0	28.2 ± 5.2*
Body fat, %	21.0 ± 5.5	31.8 ± 8.6*
Steps/day	11 059 ± 3375	10 828 ± 3717
Vector magnitude	2 377 144 ± 689 745	2 310 781 ± 772 657
Recent dietary intake^a^		
Energy intake, kcal/day	2343 ± 935	2040 ± 629
Carbohydrates, g/day	252 ± 96	232 ± 99
Fat, g/day	101 ± 51	84 ± 26
Protein, g/day	106 ± 53	84 ± 35
Sodium, mg/day	3654 ± 1571	3229 ± 1101
Sugar, g/day	103 ± 54	105 ± 65

*Note*: Values are the mean ± SD; *p < 0.05. Abbreviations: MA, middle‐aged adult with cardiometabolic disease risk factors; YA, young, healthy adult.

^a^ Dietary intake assessed during 72 h prior to experimental visit.

### 3.1 Blood glucose and insulin

Time effects were observed, whereby glucose (*p *< 0.001, η^2^ = 0.43) and insulin (*p *< 0.001, η^2^ = 0.67) were significantly higher at 30 and 60 min compared with baseline and at 30 min compared with 60 min (Table 2). There was also a significant group effect, whereby young adults had overall lower glucose compared with middle‐aged adults (*p *= 0.002, η^2^ = 0.24). There were no significant group‐by‐time interactions for changes in glucose or insulin values after meal consumption.

**TABLE 2:** Changes in glucose and insulin in young, healthy adults versus middle‐aged adults with cardiometabolic disease risk factors at baseline and at 30 and 60 min following the high‐sugar mixed meal.

Parameter	Group	BL	30 min	60 min	Group	Time	GxT
Glucose, mg/dL	YA	73.8 ± 25.2	100.9 ± 16.0	81.1 ± 13.2	0.002 (0.24)	<0.001 (0.43)^a^, ^b^, ^c^	0.18 (0.04)
	MA	86.5 ± 6.7	109.9 ± 15.7	100.9 ± 21.6			
Insulin, pg/mL	YA	302.6 ± 379.4	1997.6 ± 1154.1	1169.6 ± 409.7	0.74 (0.003)	<0.001 (0.67)^a^, ^b^, ^c^	0.74 (0.008)
	MA	483.5 ± 471.4	1991.3 ± 1114.4	1183.0 ± 473.0			

*Note*: Abbreviations: BL, baseline; GxT, group‐by‐time interaction; MA, middle‐aged adult with cardiometabolic disease risk factors; YA, young, healthy adult.

^a^ Time effect, *p* < 0.05 BL versus 30 min.

^b^ Time effect, *p*< 0.05 BL versus 60 min.

^c^ Time effect, *p* < 0.05 30‐ versus 60 min.

### 3.2 Blood pressure

Significant time and group effects were observed for mean arterial pressure, whereby it was trending to decrease at 30 min compared with baseline and was significantly lower in young adults (Table 3; *p *< 0.05). Significant group‐by‐time interactions were observed for carotid pulse pressure (Figure 1a; *p* < 0.01) and systolic pressure (Table 3; *p* < 0.01), whereby pulse pressure was lower at 60 min compared with 30 min in young adults, and both pulse and systolic pressure were lower at 60 min compared with baseline in middle‐aged adults.

**TABLE 3:** Changes in blood pressure, heart rate and large artery stiffness in young, healthy adults versus middle‐aged adults with cardiometabolic disease risk factors at baseline and at 30 and 60 min following the high‐sugar mixed meal.

Parameter	Group	BL	30 min	60 min	Group	Time	GxT
Mean arterial pressure, mmHg	YA	83 ± 8	82 ± 7	81 ± 8	0.007 (0.17)	0.01 (0.10)	0.15 (0.05)
	MA	93 ± 13	89 ± 11	89 ± 11			
Carotid systolic pressure, mmHg	YA	105 ± 12	108 ± 11	105 ± 9	0.048 (0.097)	0.046 (0.08)	0.003 (0.14)
	MA	119 ± 19^†^	113 ± 15^*^	112 ± 14^*^			
Heart rate, beats/min	YA	56 ± 9	63 ± 11	65 ± 11	0.67 (0.005)	<0.001 (0.56)^a^, ^b^, ^c^	0.16 (0.05)
	MA	59 ± 7	63 ± 7	65 ± 8			
cfPWV, cm/s	YA	527 ± 87	516 ± 81	515 ± 84	<0.001 (0.46)	0.08 (0.06)	0.67 (0.01)
	MA	753 ± 187	729 ± 164	742 ± 178			
Carotid β‐stiffness, a.u.	YA	4.10 ± 0.90	3.89 ± 0.92	3.82 ± 0.93	<0.001 (0.73)	0.004 (0.14)^b^	0.16 (0.05)
	MA	7.81 ± 1.44	7.12 ± 1.73	6.73 ± 1.54			

Abbreviations: BL, baseline; cfPWV, carotid–femoral pulse wave velocity; GxT, group‐by‐time interaction; MA, middle‐aged adult with cardiometabolic disease risk factors; YA, young, healthy adult.

* *p* < 0.05 versus within‐group baseline.

^†^ *p* < 0.05 versus YA within‐time point.

^a^ Time effect, *p* < 0.05 BL versus 30 min.

^b^ Time effect, *p* < 0.05 BL versus 60 min.

^c^ Time effect, *p* < 0.05 30 versus 60 min.

### 3.3 Large artery stiffness and characteristic impedance

Group effects for both carotid (*p *< 0.001) and aortic (*p *< 0.001) stiffness existed, whereby young adults had significantly lower stiffness values in comparison to middle‐aged adults (Table 3). Time effects (*p *< 0.01) were observed for carotid stiffness, which was significantly decreased at 60 min compared with baseline. Carotid characteristic impedance had significant group (*p *< 0.001) and time effects (*p *< 0.05), whereby young adults had significantly lower values than middle‐aged adults and there was a trend for lower impedance at 60 min compared with baseline (Figure 1c; Table S1). There were no significant interaction effects for large artery stiffness or characteristic impedance.

### 3.4 Carotid haemodynamics

Group effects were observed for carotid PI (*p* < 0.001) and systolic velocity (*p* < 0.05), whereby young adults had significantly higher values compared with middle‐aged adults (Table 4). Time effects (*p* < 0.001) were present for carotid artery diameter, which was significantly increased at 60 min compared with baseline and 30 min. Carotid pulsatility and systolic velocity increased, whereas diastolic velocity decreased at 30 and 60 min compared with baseline in both groups (time effects, *p* < 0.001). There were also time effects observed for the reflection index (Table S1; *p* < 0.01), which decreased at both 30 and 60 min compared with baseline. There were no significant interaction effects for carotid artery haemodynamics (*p* = 0.07–0.59).

**TABLE 4:** Changes in carotid artery haemodynamics in young, healthy adults versus middle‐aged adults with cardiometabolic disease risk factors at baseline and at 30 and 60 min following the high‐sugar mixed meal.

Parameter	Group	BL	30 min	60 min	Group	Time	GxT
Diameter, mm	YA	5.3 ± 0.4	5.4 ± 0.4	5.5 ± 0.3	0.94 (0.07)	<0.001 (0.23) ^b^, ^c^	0.16 (0.05)
	MA	5.7 ± 0.5	5.6 ± 0.6	5.7 ± 0.5			
Pulsatility index, a.u.	YA	1.85 ± 0.44	2.20 ± 0.40	2.14 ± 0.41	<0.001 (0.46)	<0.001 (0.57)^a^, ^b^	0.59 (0.01)
	MA	1.36 ± 0.25	1.59 ± 0.34	1.54 ± 0.32			
Mean velocity, cm/s	YA	42 ± 6	41 ± 5	41 ± 5	0.77 (0.002)	0.03 (0.09)	0.17 (0.05)
	MA	43 ± 7	42 ± 6	40 ± 6			
Systolic velocity, cm/s	YA	104 ± 19	113 ± 16	113 ± 21	0.04 (0.11)	<0.001 (0.23)^a^, ^b^	0.07 (0.07)
	MA	84 ± 11	90 ± 113	86 ± 14			
Diastolic velocity, cm/s	YA	27 ± 4	24 ± 4	25 ± 4	0.86 (0.001)	<0.001 (0.33)^a^, ^b^	0.31 (0.03)
	MA	27 ± 5	25 ± 4	24 ± 4			

*Note*: Abbreviations: BL, baseline; GxT, group‐by‐time interaction; MA, middle‐aged adult with cardiometabolic disease risk factors; YA, young, healthy adult.

* *p* < 0.05 versus within‐group baseline.

^a^ Time effect, *p* < 0.05 BL versus 30 min.

^b^ Time effect, *p* < 0.05 BL versus 60 min.

^c^ Time effect, *p* < 0.05 30 versus 60 min.

### 3.5 MCA haemodynamics

The MCA PI had a significant interaction term (Figure 2; Table S1; *p *< 0.05), whereby young adults had significantly higher cerebral pulsatility at 30 and 60 min compared with baseline, with no differences among middle‐aged adults with CMD risk factors. There were no significant interactions or individual group or time effects for MCA mean velocity (Table 5). The MCA resistive index (RI) had a significant group‐by‐time effect (Table 5; *p* < 0.05), whereby young adults had a significant increase at 30 and 60 min compared with baseline. Conductance had significant group and time effects (*p* < 0.05), whereby young adults had higher values than middle‐aged adults, and it increased at 30 min compared with baseline. The MCA mean velocity/ETCO_2_ had significant group‐by‐time interactions (*p* < 0.05), whereby young adults had higher values at 30 min compared with baseline, and middle‐aged adults had lower values at each time point compared with young adults. Relative percentage change in MCA mean velocity per millimetre of mercury ETCO_2_ had significant group‐by‐time interactions (*p* < 0.01), whereby middle‐aged adults had lower values compared with young adults at 30 and 60 min.

**TABLE 5:** Changes in middle cerebral artery haemodynamics, end‐tidal CO_2_ and cerebrovascular reactivity in young, healthy adults versus middle‐aged adults with cardiometabolic disease risk factors at baseline and at 30 and 60 min following the high‐sugar mixed meal.

Parameter	Group	BL	30‐min	60‐min	Group	Time	GxT
Mean velocity, cm/s	YA	63 ± 17	65 ± 17	63 ± 16	0.16 (0.05)	0.07 (0.07)	0.96 (0.001)
	MA	56 ± 16	58 ± 14	57 ± 14			
Systolic velocity, cm/s	YA	98 ± 25	106 ± 25	102 ± 25	0.18 (0.05)	<0.001 (0.22)^a^, ^b^	0.50 (0.02)
	MA	89 ± 25	94 ± 22	91 ± 23			
Diastolic velocity, cm/s	YA	46 ± 13	45 ± 13	44 ± 12	0.17 (0.05)	0.27 (0.04)	0.34 (0.03)
	MA	40 ± 11	41 ± 10	39 ± 10			
Resistive index, a.u.	YA	0.53 ± 0.58	0.58 ± 0.06^*^	0.57 ± 0.04^*^	0.92 (0.00)	<0.001 (0.23)	0.01 (0.11)
	MA	0.56 ± 0.03	0.56 ± 0.04	0.57 ± 0.04			
Conductance, cm/s/mmHg	YA	0.78 ± 0.25	0.80 ± 0.23	0.79 ± 0.24	0.02 (0.15)	0.03 (0.09)^a^	0.53 (0.02)
	MA	0.61 ± 0.16	0.66 ± 0.15	0.63 ± 0.14			
ETCO_2_, mmHg	YA	39 ± 4	39 ± 6	39 ± 3	0.41 (0.02)	0.36 (0.03)	0.53 (0.02)
	MA	40 ± 4	40 ± 4	39 ± 4			
MeanV/ETCO_2_, cm/s/mmHg	YA	1.65 ± 0.58	1.92 ± 0.46^*^	1.75 ± 0.53	<0.001 (0.37)	0.43 (0.03)	0.03 (0.11)
	MA	1.26 ± 0.26^†^	1.16 ± 0.26^†^	1.15 ± 0.41^†^			
%∆meanV/ETCO_2_,(%/mmHg	YA	3.21 ± 0.92	3.60 ± 0.61	3.35 ± 0.78	0.04 (0.13)	0.42 (0.03)	0.01 (0.14)
	MA	3.26 ± 0.81	2.88 ± 0.98^†^	2.67 ± 0.99^†^			

*Note*: Abbreviations: BL, baseline; ET, end‐tidal; GxT, group‐by‐time interaction; MA, middle‐aged adult with cardiometabolic disease risk factors; MeanV, mean velocity; YA, young, healthy adult.

* *p* < 0.05 versus within‐group baseline.

^†^ *p* < 0.05 versus YA within time point.

^a^ Time effect, *p* < 0.05 BL versus 30 min.

^b^ Time effect, *p* < 0.05 30 versus 60 min.

## 4 DISCUSSION

In this study, we compared the acute effects of an HSMM across segments of the cerebrovasculature in healthy young adults versus middle‐aged adults with risk factors, a group at an elevated risk of later‐life cerebrovascular disease. Our main findings suggest that an HSMM increases blood glucose and insulin and cerebrovascular vasodilatation and decreases carotid artery characteristic impedance in both young healthy and middle‐aged adults with risk factors but elicits differential increases in: (1) MCA PI and RI; and (2) cerebrovascular reactivity in young adults only. In sum, these data suggest that an HSMM appears to elicit divergent changes in cerebrovascular haemodynamics marked by reductions in blood pressure, vessel stiffness and characteristic impedance but increases in cerebrovascular blood flow and pulsatility that generally appear attenuated among middle‐aged adults with CMD risk factors in comparison to young healthy adults.

Consumption of an HSMM increased both glucose and insulin and resulted in similar large extracranial artery responses in both young healthy adults and middle‐aged adults with CMD risk factors. Both groups exhibited increases in carotid artery diameter, suggesting vasodilatation in response to an HSMM, which aligns with postprandial data in the peripheral vasculature (femoral artery) (Parker et al., 2020). We also noted reductions in MAP and large artery stiffness that tended to be weakly or moderately associated in the group as a whole and were not associated with the increase in carotid diameter (see Supplemental Material). This reduction in blood pressure and large artery stiffness contrasts with previous work with hyperglycaemic hyperinsulinaemia (Horton et al., 2021) and might reflect differing approaches to induce hyperglycaemia (meal vs. intravenous induction of hyperglycaemia). Indeed, prior work suggests that the microvascular haemodynamic responses might differ based on the method of glucose administration (Roberts‐Thomson et al., 2022). Together, carotid dilatation and reductions in large artery stiffness following the HSMM combined to decrease carotid characteristic impedance in both groups, all of which would be expected to alter intracranial haemodynamics. We noted increased MCA conductance and reduced carotid wave reflection following the HSMM, which are suggestive of cerebral vasodilatation (Bleasdale et al., 2003). These data suggest that an HSMM elicits similar extracranial cerebrovascular responses in young adults and middle‐aged adults with CMD risk factors that ultimately support increases in blood flow to the intracranial cerebrovasculature.

Surprisingly, we observed divergent responses in cerebral pulsatility following the HSMM, whereby MCA pulsatility increased in young adults only. The concomitant increases in the RI suggest that the change in PI was driven primarily by increases in systolic velocity (as has been shown previously with acute hyperglycaemia) (Nowaczewska et al., 2019). Increases in systolic velocity were probably driven by a larger forward‐travelling pressure wave, which tended to increase more among the young adults versus middle‐aged adults with CMD risk factors. Considering that forward wave magnitude is highly related to left ventricular contractility (Cauwenberghs et al., 2018; Curtis et al., 2007; Fok et al., 2014), this suggests modest differences in cardiac‐generated pulsatility between groups following the HSMM (although cardiac contractility and stroke volume were not measured directly, herein). We posit cautiously that cardiac contractility and the concomitant increases in forward wave magnitude, systolic velocity and pulsatility were attenuated among middle‐aged adults with CMD risk factors owing to age‐related blunting (Minaker et al., 1982) of the hyperglycaemia and hyperinsulinaemia‐induced increases in sympathetic activation (Anderson et al., 1991; Hoffman et al., 1999), coupled with age‐related cardiac adrenergic desensitization (Christou & Seals, 2008); however, this remains to be tested directly.

The differential pulsatile haemodynamic response between groups following the HSMM might alternatively, or simultaneously, reflect changes in downstream or local cerebrovascular tone. Although cerebral pulsatility might increase secondary to downstream resistance via vasoconstriction, it is not solely a measure of downstream resistance (de Riva et al., 2012) and can also reflect local changes in vessel tone that impact arterial stiffness. Although cerebral blood flow appears stable during acute hyperglycaemia (Blazey et al., 2025), the MCA might dilate in response to hyperinsulinaemia without significantly impacting velocity (Shariffi et al., 2025). The MCA dilatation during hyperinsulinaemia might structurally stiffen the vessel wall by shifting the load burden from elastin to collagen (Shariffi et al., 2025). This stiffening would alter the local vessel Windkessel properties and shift the stroke volume distribution away from diastole and to systole (reducing diastolic and increasing systolic blood velocity). As such, our data might suggest that young healthy adults exhibit greater increases in MCA dilatation following an HSMM compared with middle‐aged adults with CMD risk factors; however, direct measurement of MCA diameter in future work is necessary to test this hypothesis. Alternatively, age‐related stiffening of cerebral arteries from degradation of elastin (Fonck et al., 2009) might render middle‐aged adults with CMD risk factors less sensitive to dilatation‐induced stiffening of the MCA, because collagen already bears more of the load within the wall, which would attenuate changes in pulsatility with local dilatation and/or distension.

We also observed that the HSMM resulted in selective increases in MCA reactivity to breath‐hold‐induced changes in CO_2_ among young, healthy adults but not in middle‐aged adults with CMD risk factors. This selective increase in cerebral reactivity among the young, healthy adults following the HSMM might partly reflect their generally higher MCA mean velocity, because the statistical effects differed slightly when expressed as the percentage change in mean velocity, although the overall responses following the HSMM still differed between groups. This modest but apparent differential change in reactivity might reflect slightly greater vasoconstriction downstream of the MCA among young adults that would increase resistance and MCA PI (as discussed previously) while simultaneously increasing the hyperaemic reserve to vasodilatory stimuli. Differences in cerebral reactivity following the HSMM between young healthy and middle‐aged adults with CMD risk factors might, alternatively, reflect age‐related changes in sensitivity to vasodilatory stimuli following acute meal challenges (Marley et al., 2017). Ultimately, data examining changes in cerebral reactivity following an acute meal [high‐salt (Migdal et al., 2020), high‐fat (Patik et al., 2018; Marley et al., 2017) or mixed (Dunn & Walters, 2014) meals] or hyperglycaemic challenges (Blazey et al., 2025) are mixed, with some noting no effects (Blazey et al., 2025; Dunn & Walters, 2014; Migdal et al., 2020; Patik et al., 2018) or select reductions in reactivity (Marley et al., 2017). It is not clear whether these mixed findings reflect differential sensitivity to different types of acute meals or differing approaches to assess cerebral reactivity. This is similar to findings in the periphery, where changes in endothelial function following an acute high‐sugar stimulus are variable, with some seeing increases (Horton et al., 2022), decreases (Loader et al., 2017; Šorli & Lenasi, 2023; Varsamis et al., 2018; Weiss et al., 2008; Williams et al., 1998) or vascular segment‐specific responses (Loader et al., 2015). The heterogeneity of these findings in the literature makes it difficult to identify adequately whether responses differ between the peripheral vasulature and cerebrovasculature and should be examined simultaneously in future work.

The complex cerebrovascular response to an HSMM across extra‐ and intracranial cerebrovasculature and young, healthy adluts versus middle‐aged adults with CMD risk factors complicates the implications of these data. We observed increased vasodilatation, cerebral reactivity and MCA pulsatility in response to an HSMM, similar to segment‐specific observations in the peripheral vasculature (conduit artery vasodilatation paired with reductions in microvascular flow) (Parker et al., 2020) following hyperglycaemic challenges. Moreover, these divergent changes appeared to occur selectively among the younger adults, not among middle‐aged adults with CMD risk factors. The absence of a clear detrimental response to an HSMM among middle‐aged adults with CMD risk factors is likely not to be indicative of ‘protection’ against an HSMM, but might simply reflect greater baseline vascular dysfunction that attenuates reactivity to a given perturbation, similar to the loss of peripheral vascular reactivity to acute inflammation seen with advancing age (Lefferts & Ranadive, 2024). Ultimately, these findings might suggest that the intracranial cerebral haemodynamic response to an acute HSMM is more sensitive to age and CMD risk factors than the extracranial cerebrovasculature. These results highlight a complex cerebrovascular response to an acute HSMM challenge that differs in young healthy adults versus middle‐aged adults with CMD risk factors. These differential responses might accumulate in differential changes over time when repeatedly consumed as part of a Western diet; however, future work is necessary to test this hypothesis directly, in addition to its potential implications for links between diet and cerebrovascular health.

### 4.1 Limitations

In this study, we compared cerebrovascular responses to an HSMM in middle‐aged adults with CMD risk factors versus young healthy adults as a means of comparing a vulnerable population with a non‐vulnerable population and maximizing the ability to identify differential responses within this early‐stage research. This design consideration means these data do not reflect the independent effects of ageing or CMD risk factor burden on cerebrovascular responses to a HSMM or their implications for cerebrovascular health. The standardized meal in this study successfully increased blood glucose and insulin levels as intended but was not tailored relative to individual body size, which can impact how meals are metabolized and thus could alter the haemodynamic response to the meal. Diet recall was conducted during the 72 h leading up to the acute meal study visit, and thus was not standardized for the number of weekdays or weekends captured within the recall. The purpose of this dietary recall was to quantify the type of diet consumed immediately prior to the acute meal challenge, rather than to assess the overall diet composition of individuals or the intake that would require a more representative sample of days.

This study lacks cerebrovascular assessment of intracranial haemodynamics downstream of the MCA (e.g., cerebral small artery or microvascular haemodynamics) that, to date, remain inherently challenging, costly (e.g., four‐dimensional flow MRI) and not without their own limitations (e.g., near‐infrared spectroscopy). Instead, this study relied on comprehensive assessment of upstream measures of haemodynamics (e.g., characteristic impedance, wave transmission/reflection via wave separation analysis) that have been associated with changes in downstream intracranial cerebral haemodynamics. Nonetheless, direct assessment of small artery and downstream microvascular responses to an HSMM in young, healthy adults and middle‐aged adults with CMD risk factors is necessary to provide direct support to some of the hypothesized vascular mechanisms underlying the differential pulsatile haemodynamic responses observed herein. Carotid characteristic impedance was derived as the ratio between peak derivatives of carotid pressure and flow (Reed et al., 2024), which might differ in comparison to other approaches (pressure‐blood velocity loop and Fourier methods). Future work should examine the influence of repeatedly consuming meals like this over a short period of time (10–14 days), which might elicit more substantial cerebrovascular perturbations that provide better evidence of the influence of repeated high‐sugar, mixed‐meal composition on changes in cerebrovascular haemodynamics that might contribute to cerebrovascular disease risk among groups that regularly consume this type of diet.

## 5 CONCLUSION

Our data suggest that the complex cerebrovascular haemodynamic response to an HSMM appears to differ between: (1) segments of the extra‐ versus intravascular cerebrovascular tree; and (2) young, healthy adults and middle‐aged adults with CMD risk factors. Differential increases in MCA pulsatility among young, healthy adults in comparison to middle‐aged adults with CMD risk factors following an HSMM might stem from modest differences in the effect of the HSMM on extracranial generation and propagation of pulsatile haemodynamics, combined with larger group differences in intracranial cerebrovascular responses to the HSMM that differentially impact local dilatation at the MCA and downstream cerebrovascular tone.

## AUTHOR CONTRIBUTIONS

Wesley K. Lefferts, Rudy J. Valentine and Krista S. Reed conceived and designed the research. Wesley K. Lefferts, Krista S. Reed, Molly R. Crew and Abby M. Frescoln collected the data. Wesley K. Lefferts, Krista S. Reed, Molly R. Crew, Abby M. Frescoln and Spencer M. Romanowski analysed the data. Wesley K. Leffert, Rudy J. Valentine and Krista S. Reed interpreted results of experiments and trial. Wesley K. Lefferts, Krista S. Reed and Molly R. Crew prepared figures. Wesley K. Lefferts, Krista S. Reed and Molly R. Crew drafted the manuscript. Wesley K. Lefferts, Rudy J. Valentine, Krista S. Reed, Molly R. Crew, Abby M. Frescoln and Spencer M. Romanowski edited and revised manuscript. All authors approved the final version of manuscript and agree to be accountable for all aspects of the work in ensuring that questions related to the accuracy or integrity of any part of the work are appropriately investigated and resolved. All persons designated as authors qualify for authorship, and all those who qualify for authorship are listed.

## CONFLICT OF INTEREST

No conflicts of interest, financial or otherwise, are declared by the authors.

## FUNDING INFORMATION

No funding was received for this study.

## Supporting information



Supporting Information

## Data Availability

The raw data supporting the conclusions of this manuscript are available from the corresponding author upon reasonable request.
