# Supplementary material for: Effects of a high‐sugar mixed meal on cerebrovascular haemodynamics in young, healthy versus middle‐aged adults with cardiometabolic risk factors
Source: Exp Physiol. 2025 Nov 22;111(4):2370–83. doi: 10.1113/EP093238 (PMC13140483; doi:10.1113/EP093238)
Supplement: Supplementary file 1 — Supporting Information [file EPH-111-2370-s001.docx]

Methods

*At-home physical activity and dietary monitoring*

Participants wore a wrist-worn actigraphy monitor (Actigraph, wGT3X-BT, Pensacola, FL) for the 3 consecutive days leading up to the experimental visit to track physical activity. Participants were told to wear the device continuously unless interacting with water (e.g., showering, swimming). Steps were calculated using the device’s onboard software. Daily steps and vector magnitude were averaged across the 3 days for analysis. Participants also completed 24-hour diet recalls (ASA24) for the same 3 days as the wrist-worn activity monitor. Participants were told to recall all food, fluid, and supplement consumption, time of consumption, and amount consumed. Total daily caloric, carbohydrate, fat, protein, sodium, and sugar intake were calculated for analysis.

| Supplemental Results  **Table S1.** Middle cerebral artery pulsatility and carotid artery pulse pressure and wave dynamics in young, healthy adults versus middle-aged adults with cardiometabolic disease risk factors at baseline and 30-, and 60-min following the high-sugar mixed-meal. | | | | | | | |  |
| --- | --- | --- | --- | --- | --- | --- | --- | --- |
|  | Group | BL | 30-min | 60-min | Group | Time | GxT |  |
| *Middle cerebral artery* |  |  |  |  |  |  |  |  |
| Pulsatility index | YA | 0.83 ± 0.14 | 0.96 ± 0.15* | 0.93 ± 0.11* | 0.99 (0.001) | <0.001 (0.22) | 0.01 (0.11) |  |
| (au) | MA | 0.89 ±0.09 | 0.91 ± 0.10 | 0.91 ± 0.10 |  |  |  |  |
| *Carotid artery* |  |  |  |  |  |  |  |  |
| Pulse pressure | | YA | 39 ± 8 | 42 ± 9 | 39 ± 7^Ϯ^ | 0.61 (0.007) | 0.048 (0.08) | 0.002 (0.15) |
| (mmHg) | | MA | 44 ± 12 | 41 ± 8 | 39 ± 8* |  |  |  |
| Characteristic impedance | YA | 3739 ± 1285 | 3381 ± 1094 | 3259 ± 934 | 0.01 (0.15) | 0.04 (0.08) | 0.93 (0.002) |  |
| (dyne∙s/cm^5^) | MA | 4630 ± 1818 | 4130 ± 1156 | 4184 ± 1090 |  |  |  |  |
| Forward wave magnitude | YA | 31 ± 8 | 35 ± 8 | 33 ± 6 | 0.87 (0.001) | 0.27 (0.03) | 0.09 (0.06) |  |
| (mmHg) | MA | 33 ± 9 | 32 ± 7 | 32 ± 6 |  |  |  |  |
| Backward wave magnitude | YA | 15 ± 3 | 15 ± 4 | 14 ± 3 | 0.97 (0.00) | 0.06 (0.07) | 0.59 (0.01) |  |
| (mmHg) | MA | 16 ± 5 | 15 ± 3 | 14 ± 4 |  |  |  |  |
| Reflection index | YA | 0.50 ± 0.06 | 0.45 ± 0.08 | 0.44 ± 0.07 | 0.97 (0.00) | 0.005 (0.13)^a,b^ | 0.28 (0.03) |  |
| (au) | MA | 0.48 ± 0.11 | 0.46 ± 0.09 | 0.45 ± 0.08 |  |  |  |  |
| MCA, middle cerebral artery; CCA, common carotid artery; BL, baseline; YA, young, healthy adult; MA, middle-aged adult with cardiometabolic disease risk factors; GxT, group-by-time interaction.*p<0.05 vs within-group baseline. ^Ϯ^p<0.05 vs within-group 30-min. ^a^time effect, p<0.05 BL vs 30-min, ^b^time effect, p<0.05 BL vs 60-min. | | | | | | | |  |

| **Table S2**. Correlations between changes in stiffness, mean arterial pressure, and carotid diameter in entire sample (n=41); reported as correlation coefficient (p-value). | | | | |
| --- | --- | --- | --- | --- |
|  | ∆cfPWV | ∆β-stiffness | ∆MAP | ∆CCA diameter |
| ∆cfPWV |  | ***0.33***  ***(0.04)*** | *0.23*  *(0.14)* | ***0.37***  ***(0.02)*** |
| ∆β-stiffness | 0.90  (0.57) |  | *0.16*  *(0.33)* | *0.25*  *(0.12)* |
| ∆MAP | **0.41**  **(0.007)** | 0.24  (0.14) |  | ***0.48***  ***(0.002)*** |
| ∆Car diameter | 0.24  (0.14) | 0.19  (0.26) | 0.24  (0.13) |  |
| cfPWV, carotid femoral pulse wave velocity; MAP, mean arterial pressure; Car, carotid; **Bold** denotes significant correlations. Black text (lower portion of matrix) reports correlation with change from baseline to 30-min timepoint; *grey italics* text (upper portion of matrix) reports correlation with change from baseline to 60-minute timepoints. | | | | |
| **Table S3**. Correlations between changes in stiffness, mean arterial pressure, and carotid diameter in young adults (n=21); reported as correlation coefficient (p-value). | | | | |
|  | ∆cfPWV | ∆β-stiffness | ∆MAP | ∆CCA diameter |
| ∆cfPWV |  | *-0.09*  *(0.72)* | *0.02*  *(0.94)* | *0.19*  *(0.40)* |
| ∆β-stiffness | -0.098  (0.67) |  | *0.15*  *(0.52)* | *0.16*  *(0.48)* |
| ∆MAP | 0.19  (0.42) | 0.23  (0.32) |  | ***0.57***  ***(0.007)*** |
| ∆Car diameter | 0.09  (0.69) | 0.04  (0.87) | 0.14  (0.55) |  |
| cfPWV, carotid femoral pulse wave velocity; MAP, mean arterial pressure; Car, carotid; **Bold** denotes significant correlations. Black text (lower portion of matrix) reports correlation with change from baseline to 30-min timepoint; *grey italics* text (upper portion of matrix) reports correlation with change from baseline to 60-minute timepoints. | | | | |
| **Table S4**. Correlations between changes in stiffness, mean arterial pressure, and carotid diameter in middle-aged adults with risk factors (n=20); reported as correlation coefficient (p-value). | | | | |
|  | ∆cfPWV | ∆β-stiffness | ∆MAP | ∆CCA diameter |
| ∆cfPWV |  | *0.46*  *(0.06)* | *0.41*  *(0.07)* | ***0.52***  ***(0.02)*** |
| ∆β-stiffness | 0.11  (0.67) |  | *0.14*  *(0.59)* | *0.26*  *(0.30)* |
| ∆MAP | **0.48**  **(0.03)** | 0.20  (0.43) |  | *0.35*  *(0.13)* |
| ∆Car diameter | 0.26  (0.27) | 0.21  (0.40) | 0.22  (0.36) |  |
| cfPWV, carotid femoral pulse wave velocity; MAP, mean arterial pressure; Car, carotid; **Bold** denotes significant correlations. Black text (lower portion of matrix) reports correlation with change from baseline to 30-min timepoint; *grey italics* text (upper portion of matrix) reports correlation with change from baseline to 60-minute timepoints. | | | | |
